# Supplementary material for: All-fiber tribo-ferroelectric synergistic electronics with high thermal-moisture stability and comfortability
Source: Nat Commun. 2019 Dec 5;10:5541. doi: 10.1038/s41467-019-13569-5 (PMC6895236; doi:10.1038/s41467-019-13569-5)
Supplement: Supplementary file 1 — Supplementary Information [file 41467_2019_13569_MOESM1_ESM.pdf]

Supplementary Information

All-fiber tribo-ferroelectric synergistic electronics with high thermal-moisture stability and comfortability

Weifeng Yang[1†], Wei Gong[1†], Chengyi Hou[1]*, Yun Su[2], Yinben Guo[1], Wei Zhang[1], Yaogang Li[3], Qinghong Zhang[3]*, Hongzhi Wang[1]*

†These authors contributed equally to this work.

*Corresponding author: Chengyi Hou (hcy@dhu.edu.cn), Qinghong Zhang (zhangqh@dhu.edu.cn), Hongzhi Wang (wanghz@dhu.edu.cn)

This file includes:

Supplementary Figures

Supplementary Figure 1. Effect of electrospinning parameters on output performance.

Supplementary Figure 2. Effect of electrospinning parameters on nanofibers.

Supplementary Figure 3. Hysteresis loop test procedure for ferroelectric P(VDF-TrFE) nanofibers.

Supplementary Figure 4. The electrical test of the e-textile in experiment.

Supplementary Figure 5. Synergistic mechanism in single ferroelectric polarized e-textile.

Supplementary Figure 6. Effect of primary polarization direction of P(VDF-TrFE) on output performance.

Supplementary Figure 7. Effect of inner/outer ferroelectric layer thickness on output performance.

Supplementary Figure 8. Depolarization of electrospun P(VDF-TrFE) nanofiber nonwovens.

Supplementary Figure 9. Comparison of output performance of NP, SFP and DFP e-textiles.

Supplementary Figure 10. Working mechanism of the tribo-ferroelectric synergistic electrics.

Supplementary Figure 11. Microphotographs, contact angles and infrared characterization of electronics.

Supplementary Figure 12. Effect of pore size and thickness of fiber layer on water evaporation rate.

Supplementary Figure 13. Test method of breathability, moisture permeability, water evaporation rate.

Supplementary Figure 14. Principle of thermal resistance and evaporative resistance test.

Supplementary Figure 15. Simulation test for different sweating levels of the human body.

Supplementary Figure 16. Cyclic data of voltage and humidity of e-textiles during moisture wicking.

Supplementary Figure 17. Washing durability test of the e-textile.

Supplementary Figure 18. Electrical output performance of the e-textiles.

Supplementary Figure 19. Detailed description of the application of the electronics.

Supplementary Figure 20. The characterization of pressure sensing by free falling impact test.

Supplementary Figure 21. The Self-charging, self-sensing monitoring system for capturing human movement.

Supplementary Tables

Supplementary Table 1. Summary of measurement conditions and electrical parameters of published triboelectric textiles.

Supplementary Table 2. Statistical summary table of relevant parameters in the free fall impact test.

Supplementary Notes

Supplementary Note 1. The effect of primary polarization direction (θ) and thickness (D_{SFP}) of ferroelectricity on the performance of SFP e-textile.

Supplementary Note 2. Depolarization process of electrospun P(VDF-TrFE) nanofiber nonwovens.

Supplementary Note 3. Comparison of electrical properties of UP, SFP and DFP e-textiles to verify the tribo-ferroelectric synergistic mechanism.

Supplementary Note 4. Detailed description of the tribo-ferroelectric synergistic mechanism in DFP e-textiles.

Supplementary Note 5. Effect of PA6 layer thickness on water evaporation rate of moisture wicking fabric.

Supplementary Note 6. Thermal resistance and evaporative resistance test methods and calculation formulas for functional textiles.

Supplementary Note 7. Cyclic test of electrical output and surface relative humidity of e-textiles during multiple moisture wicking.

Supplementary Note 8. Description of the washing test.

Supplementary Note 9. Interpretation of the comparison method of output power density in triboelectric textiles.

Supplementary Note 10. Free falling impact test proves that e-textile has certain pressure sensing characteristics.

Supplementary Note 11. Interpretation of signal processing, real-time mapping, and foot pressure sensing in self-charging, self-sensing gesture monitoring system.

Supplementary Figures

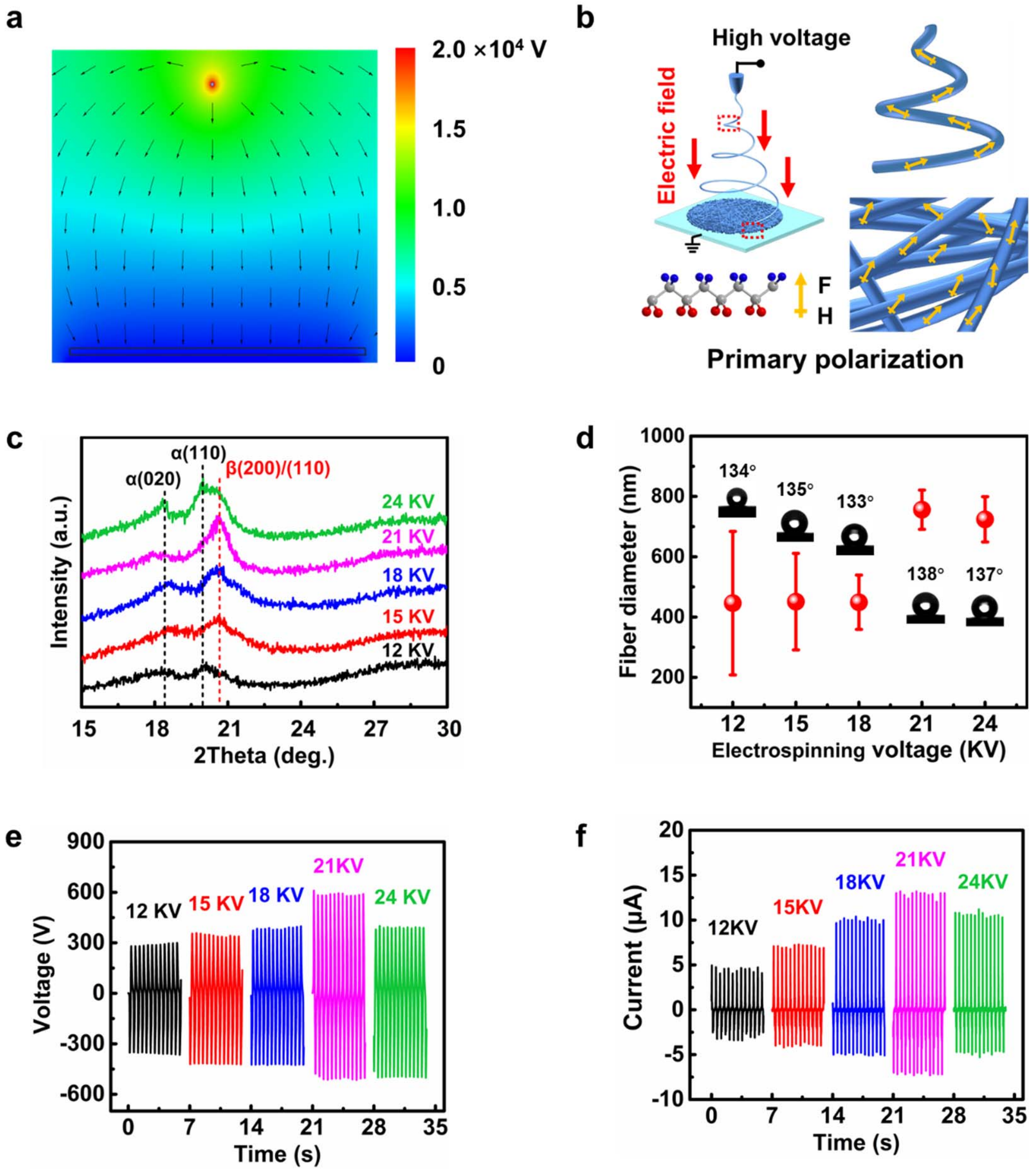

Supplementary Figure 1. Effect of electrospinning parameters on output performance. (a) The ANSYS simulation of electrostatic field intensity and direction under electrospinning. **(b)** The CF_2 dipole of P(VDF-TrFE) is oriented during the formation of nanofibers (primary polarization) under electrospinning. **(c)** XRD patterns indicate that the contents of α phase and β phase in P(VDF-TrFE) nanofibers are different by applying different electrospinning voltages. When voltage is 21 kV, the characteristic peak of β phase (20.6°) is the strongest and the sample has the most β phase. **(d)** Diameter distribution and contact angle of P(VDF-TrFE) nanofibers prepared by different electrospinning voltages. The error bars correspond to standard deviation caused by the statistical uncertainty of measurement. **(e)**, **(f)** Effect of P(VDF-TrFE) nanofibers prepared under different spinning voltages on the performance of e-textiles.

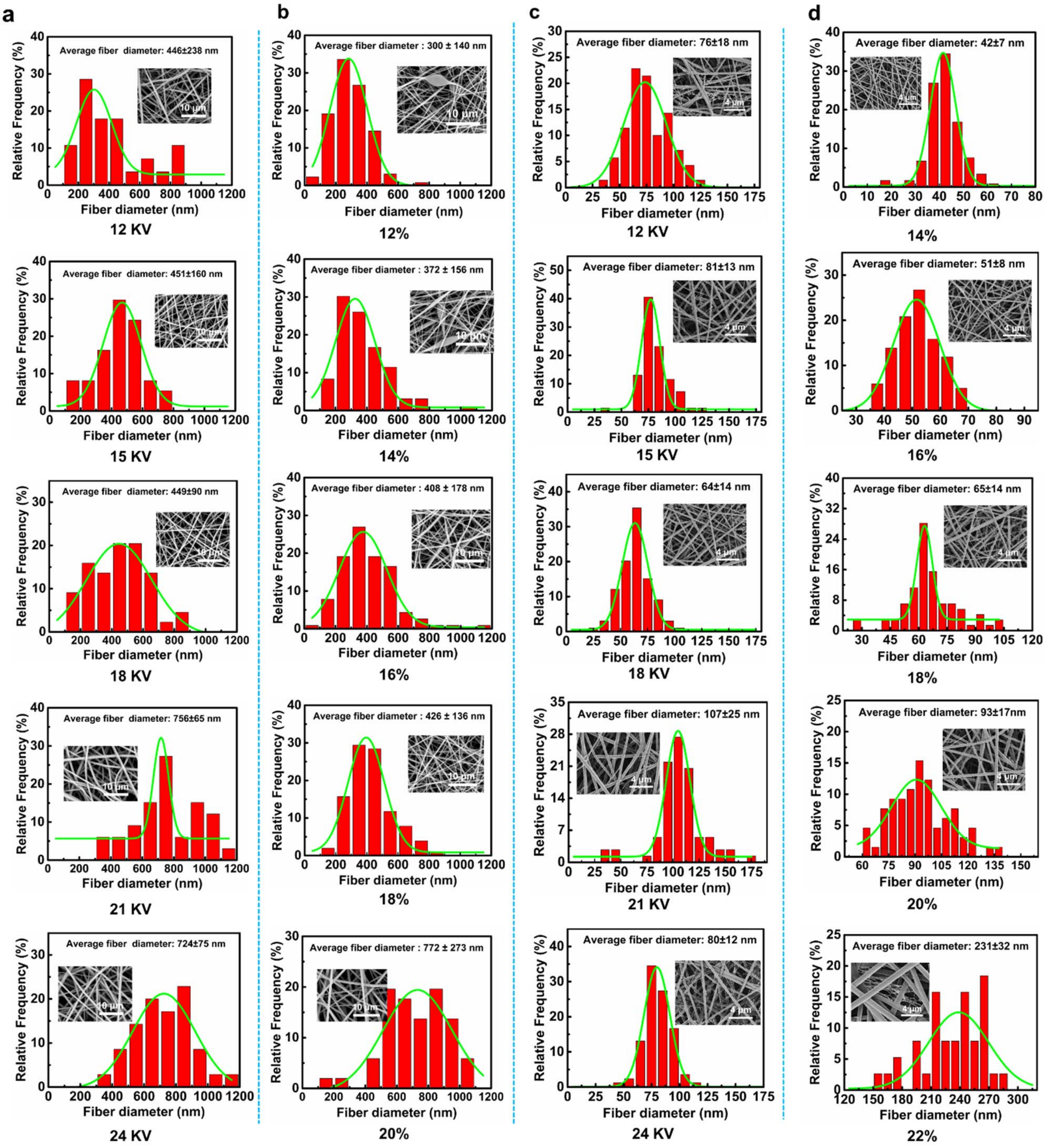

Supplementary Figure 2. Effect of electrospinning parameters on nanofibers. Effect of **(a)** electrospinning voltage and **(b)** solution concentration of P(VDF-TrFE) on morphology and diameter distribution. Effect of **(c)** electrospinning voltage and **(d)** solution concentration of PA6 on morphology and diameter distribution.

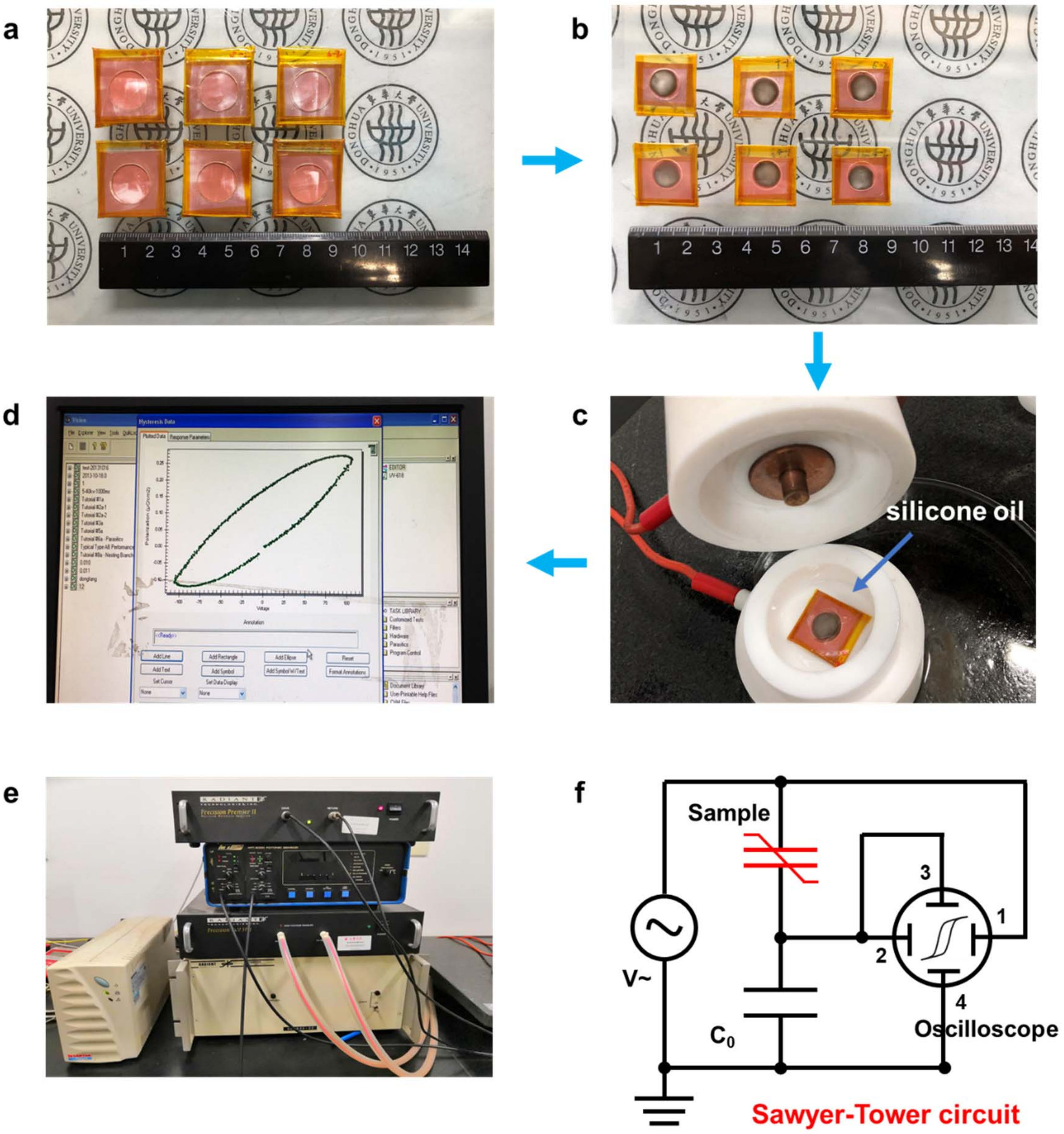

Supplementary Figure 3. Hysteresis loop test procedure for ferroelectric P(VDF-TrFE) nanofibers. (a) P(VDF-TrFE) nanofiber film is adhered to the surface of Cu foil, pressed with a 5 MPa cold press and covered with polyvinyl chloride (PVC) as a mask. **(b)** An Al electrode having a thickness of about 100 nm was deposited on the surface of P (VDF-TrFE). **(c)** P(VDF-TrFE) sample was immersed in silicone oil (preventing breakdown) for hysteresis loop test. **(d)** Photograph of hysteresis loop of P(VDF-TrFE) nanofiber sample during ferroelectric performance test. **(e)** Ferrielectric Tester (Radiant Precision Premier II). **(f)** Sawyer-Tower circuit diagram for hysteresis loop test.

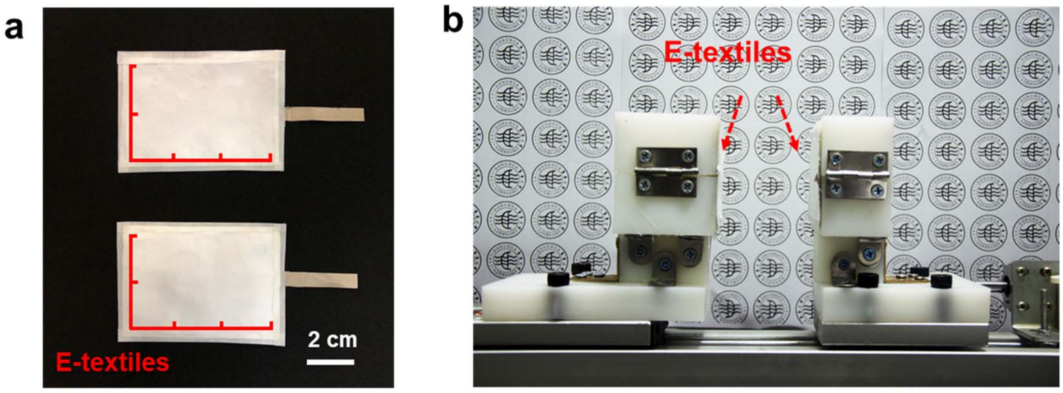

Supplementary Figure 4. The electrical test of the e-textile in experiment. (a) Digital photograph of e-textiles during test. The part marked by red ruler is the effective friction zone, and the size is 4×6 cm. **(b)** Test status of e-textiles in vertical contact mode.

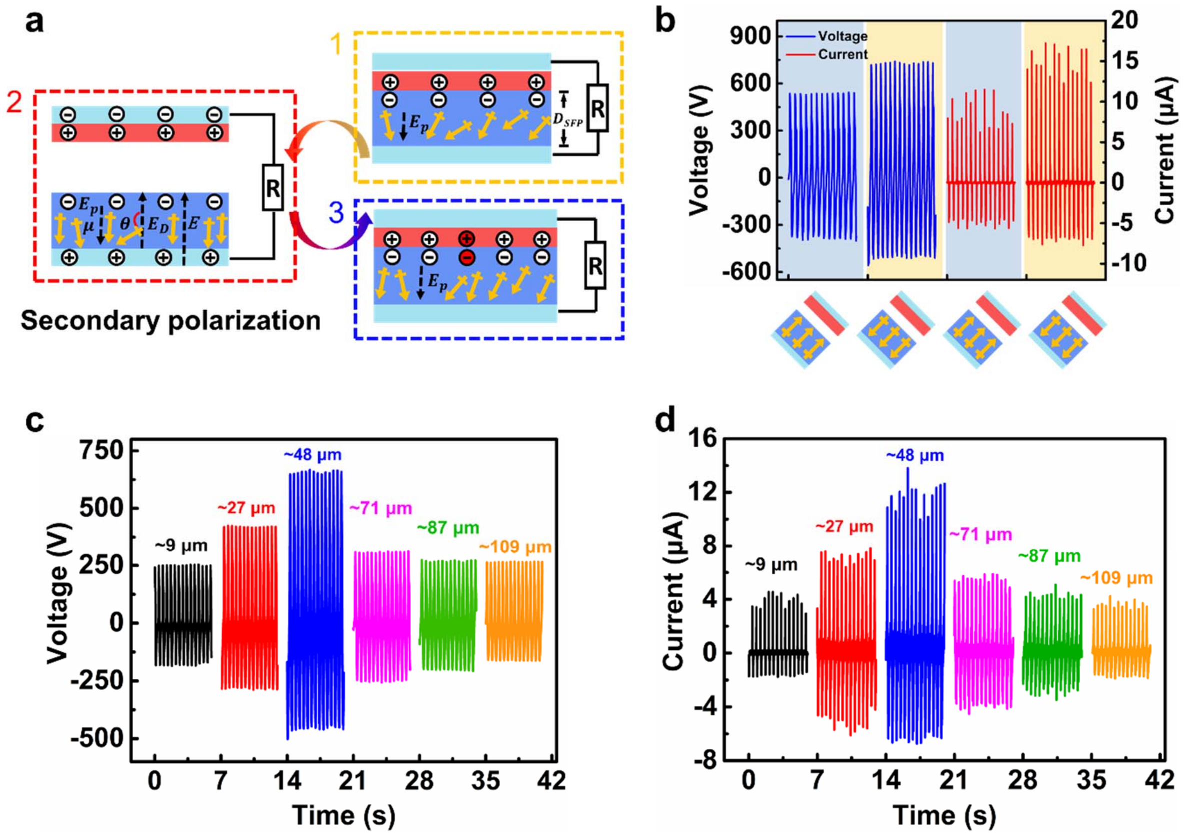

Supplementary Figure 5. Synergistic mechanism in single ferroelectric polarized e-textile. (a) Schematic diagram of tribo-ferroelectric synergistic model in single ferroelectric polarization (SFP) e-textile. **(b)** Influence of primary polarization direction (θ) of P(VDF-TrFE) nanofibers on the performance of SFP e-textile. **(c)**, and **(d)** Effect of ferroelectric layer thickness (D_{SFP}) on voltage (under 100 MΩ load) and short-circuit current of SFP e-textile.

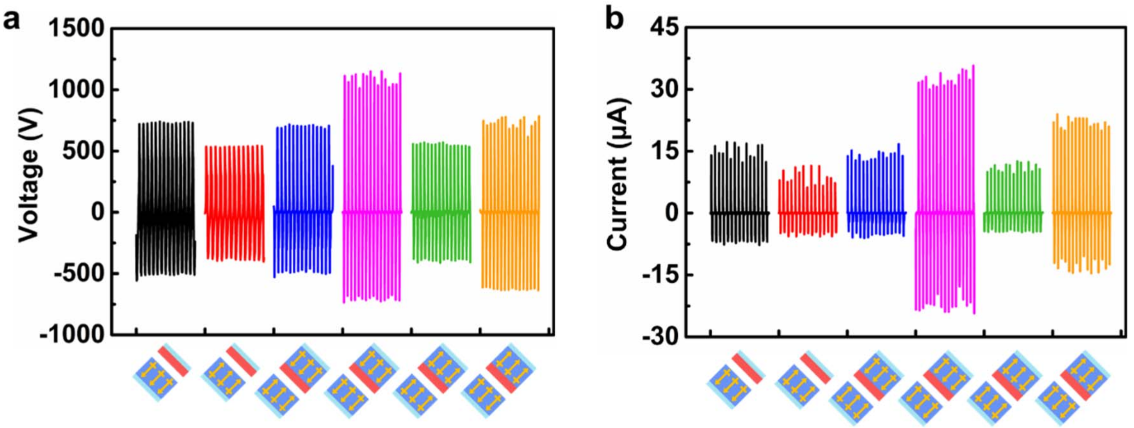

Supplementary Figure 6. Effect of primary polarization direction of P(VDF-TrFE) on output performance. (a) Voltage (under 100 MΩ load). (b) Short-circuit current.

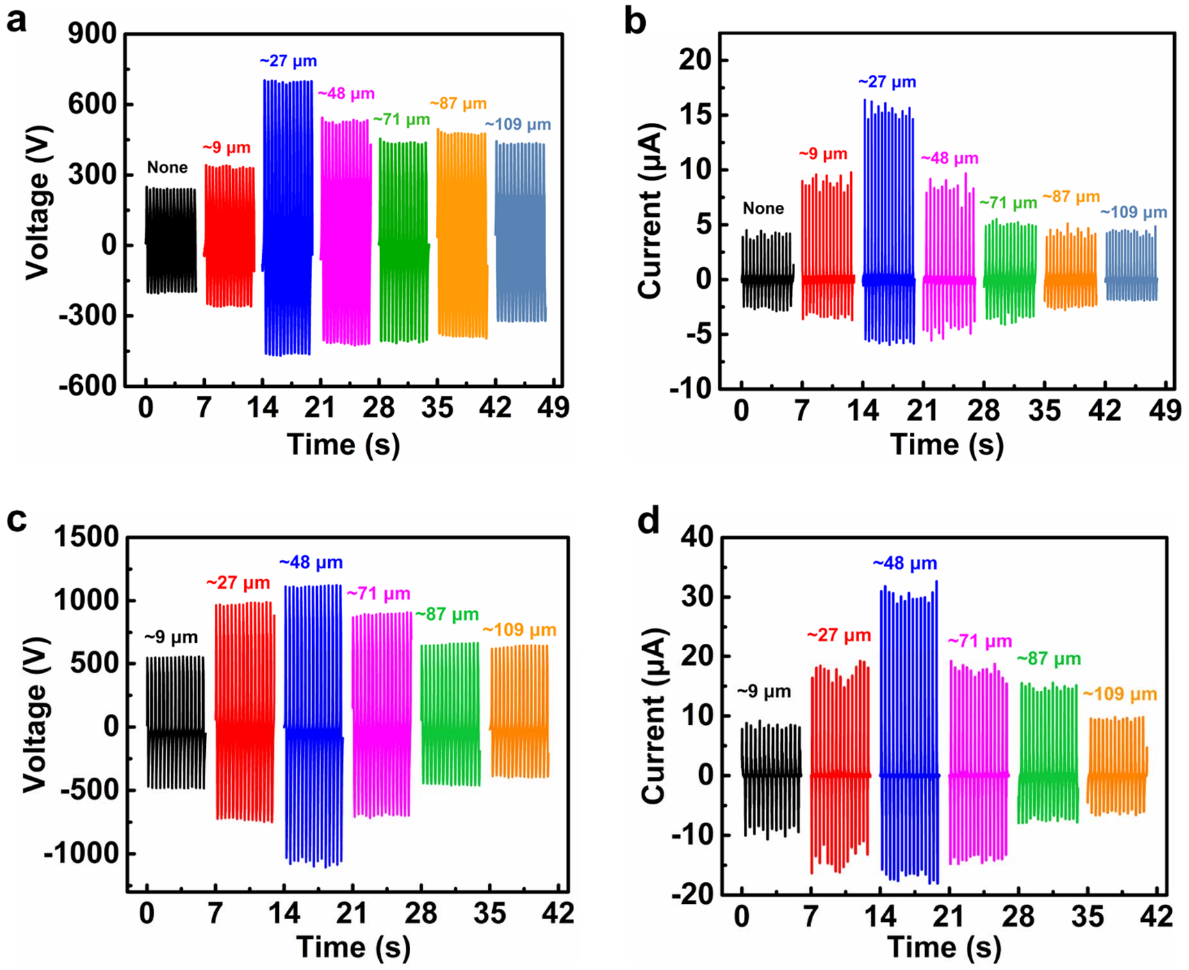

Supplementary Figure 7. Effect of inner/outer ferroelectric layer thickness on output performance. (a) and **(b)** Effect of inner ferroelectric thickness ($D_{\text{DFP-i}}$ (When $D_{\text{DFP-o}} = 87$ μm)) on voltage (under 100 MΩ load) and short-circuit current in DFP e-textile. **(c)** and **(d)** Effect of outer ferroelectric thickness ($D_{\text{DFP-o}}$ (When $D_{\text{DFP-i}} = 27$ μm)) on voltage (under 100 MΩ load) and short-circuit current in DFP e-textile.

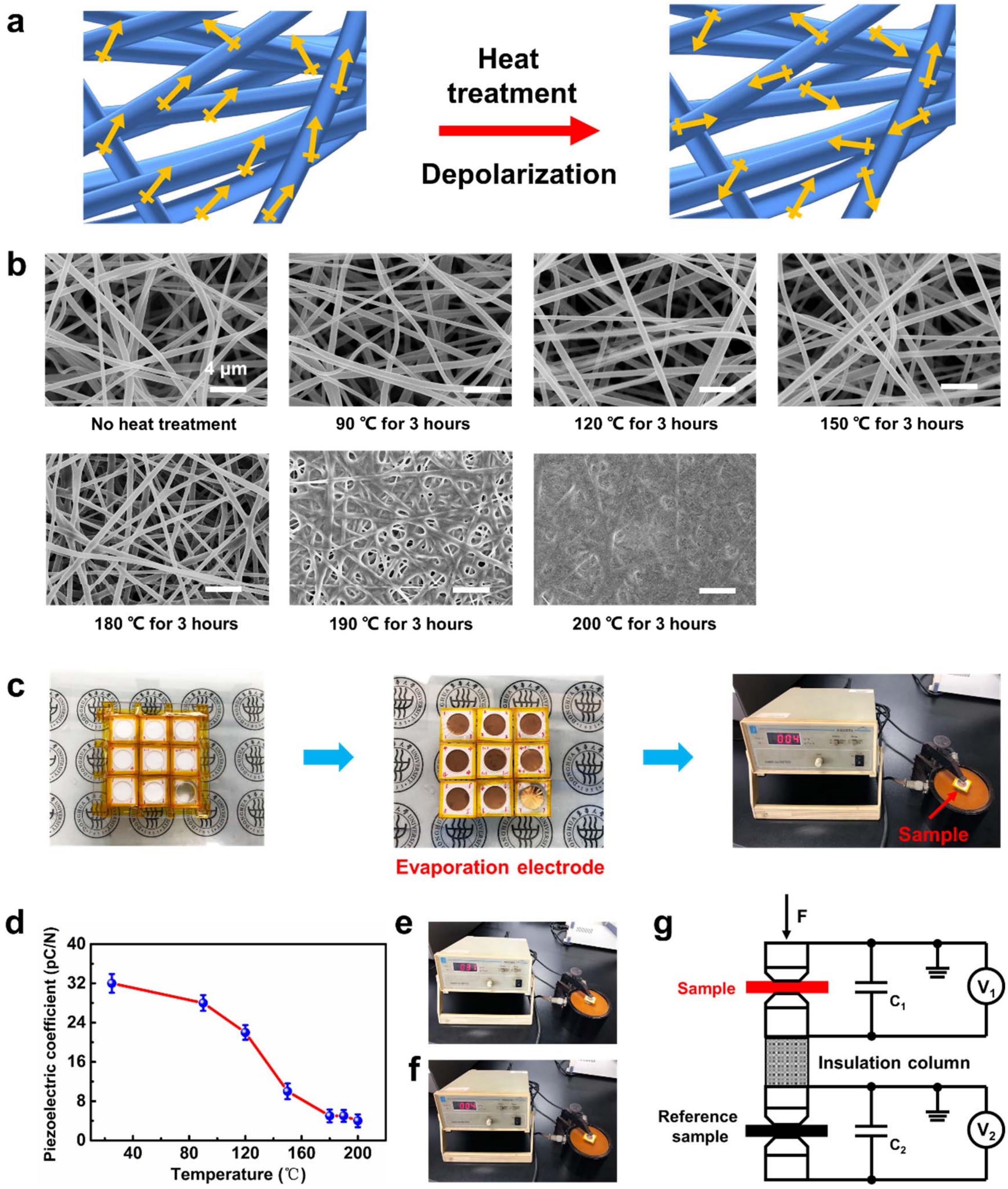

Supplementary Figure 8. Depolarization of electrospun P(VDF-TrFE) nanofiber nonwovens. (a) Schematic diagram of depolarization of P(VDF-TrFE) ferroelectricity by heat treatment. **(b)** Micrographs of P(VDF-TrFE) nanofibers after different heat treatment temperatures. **(c)** Measurement of piezoelectric coefficient (d_{33}) of depolarized P(VDF-TrFE) nanofiber nonwovens. **(d)** As the heat treatment temperature increases, the piezoelectric coefficient (d_{33}) of P(VDF-TrFE) ferroelectricity is continuously lowered to achieve the depolarization effect. Piezoelectric coefficient test of **(e)** polarized and **(f)** depolarized P(VDF-TrFE) ferroelectricity. **(g)** Circuit diagram for piezoelectric coefficient (d_{33}) test.

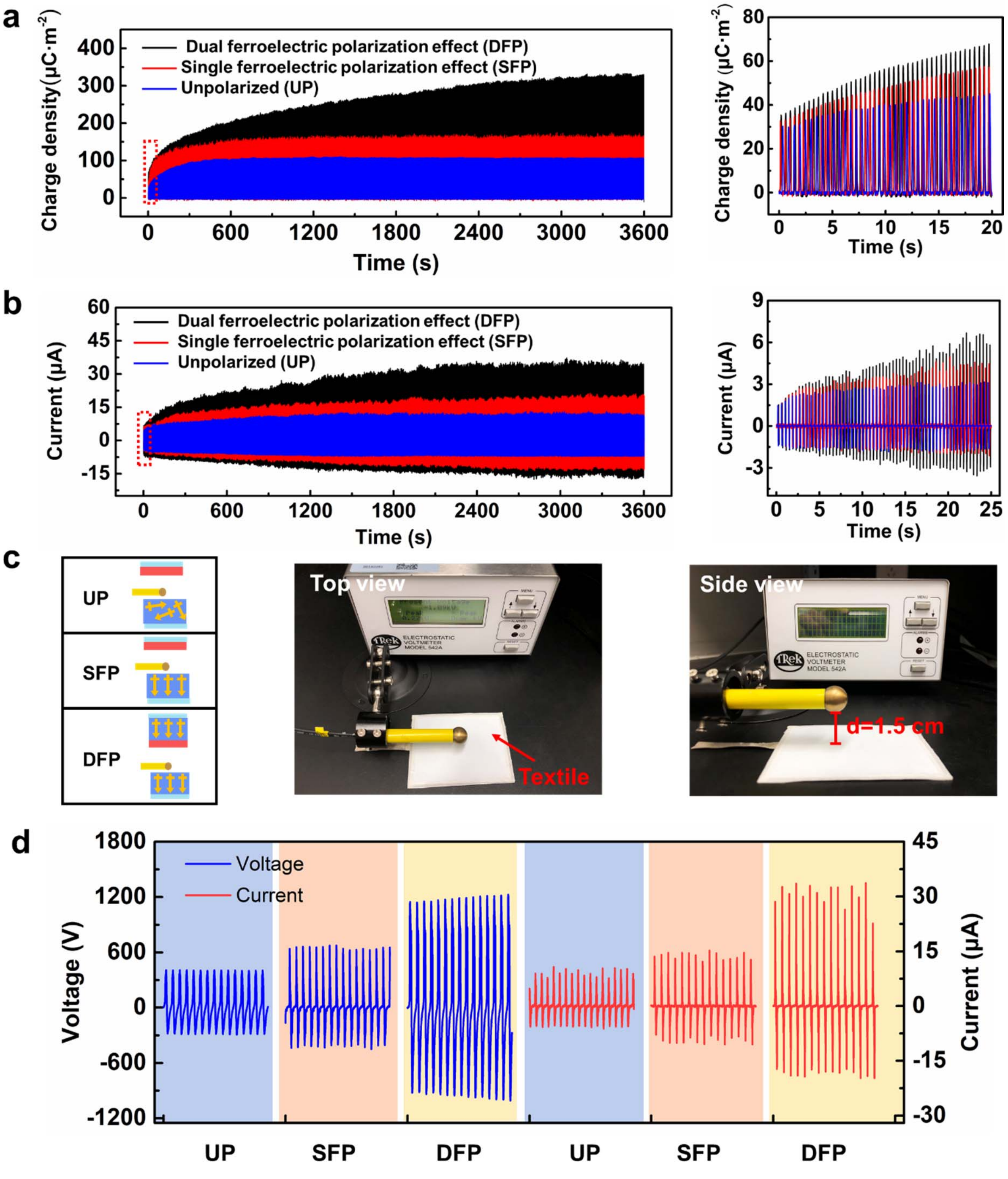

Supplementary Figure 9. Comparison of output performance of NP, SFP and DFP e-textiles. (a) The output charges density of NP, SFP and DFP e-textiles working continuously for 7200 cycles at 2.5 Hz frequency. **(b)** The output current of NP, SFP and DFP e-textiles working continuously for 7200 cycles at 2.5 Hz frequency. **(c)** Schematic diagram of the surface potential test device for textiles. **(d)** Comparison of output voltage (under 100 MΩ load) and short-circuit current of NP, SFP and DFP e-textiles at a fixed 2.5 Hz frequency.

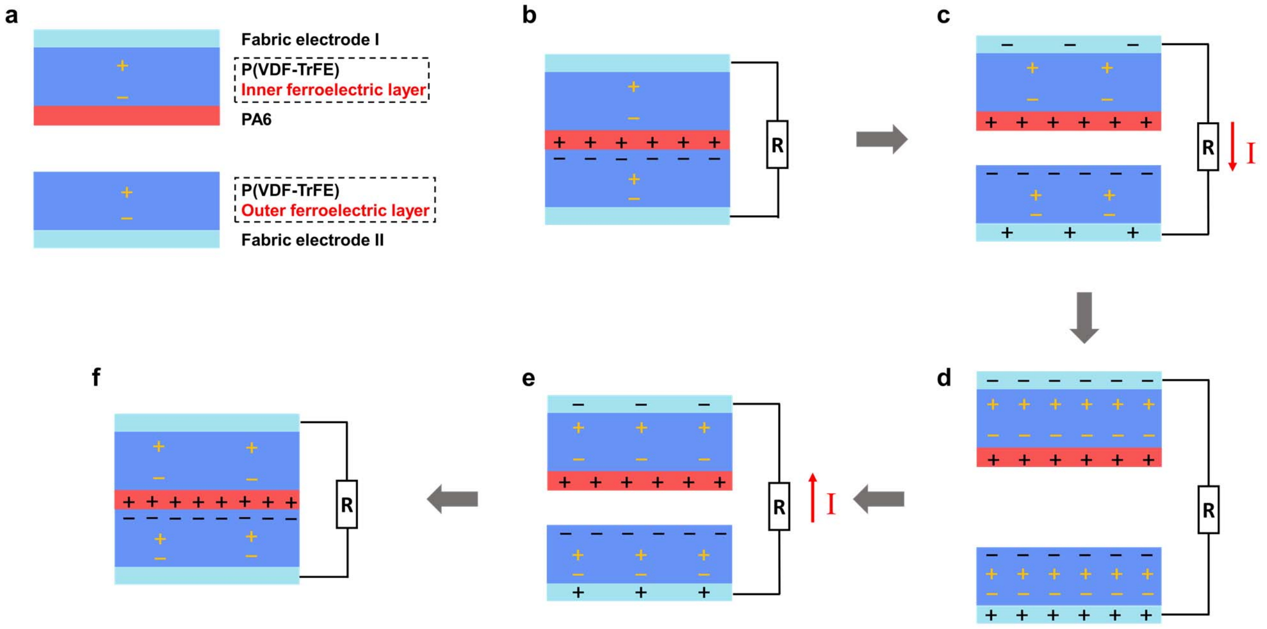

Supplementary Figure 10. Working mechanism of the tribo-ferroelectric synergistic electrics. (a) The structure diagram of the contact-separation mode e-textile. (b) In original state, two pieces of e-textile are in contact state. (c)Two pieces of e-textile begin to separate, and the distance between the two gradually increases. (d) Two pieces of e-textile separate to the maximum position and reach equilibrium. (e) Two pieces of e-textile begin to compress, and the distance between the two is gradually reduced. (f) Two pieces of e-textile are in contact with each other.

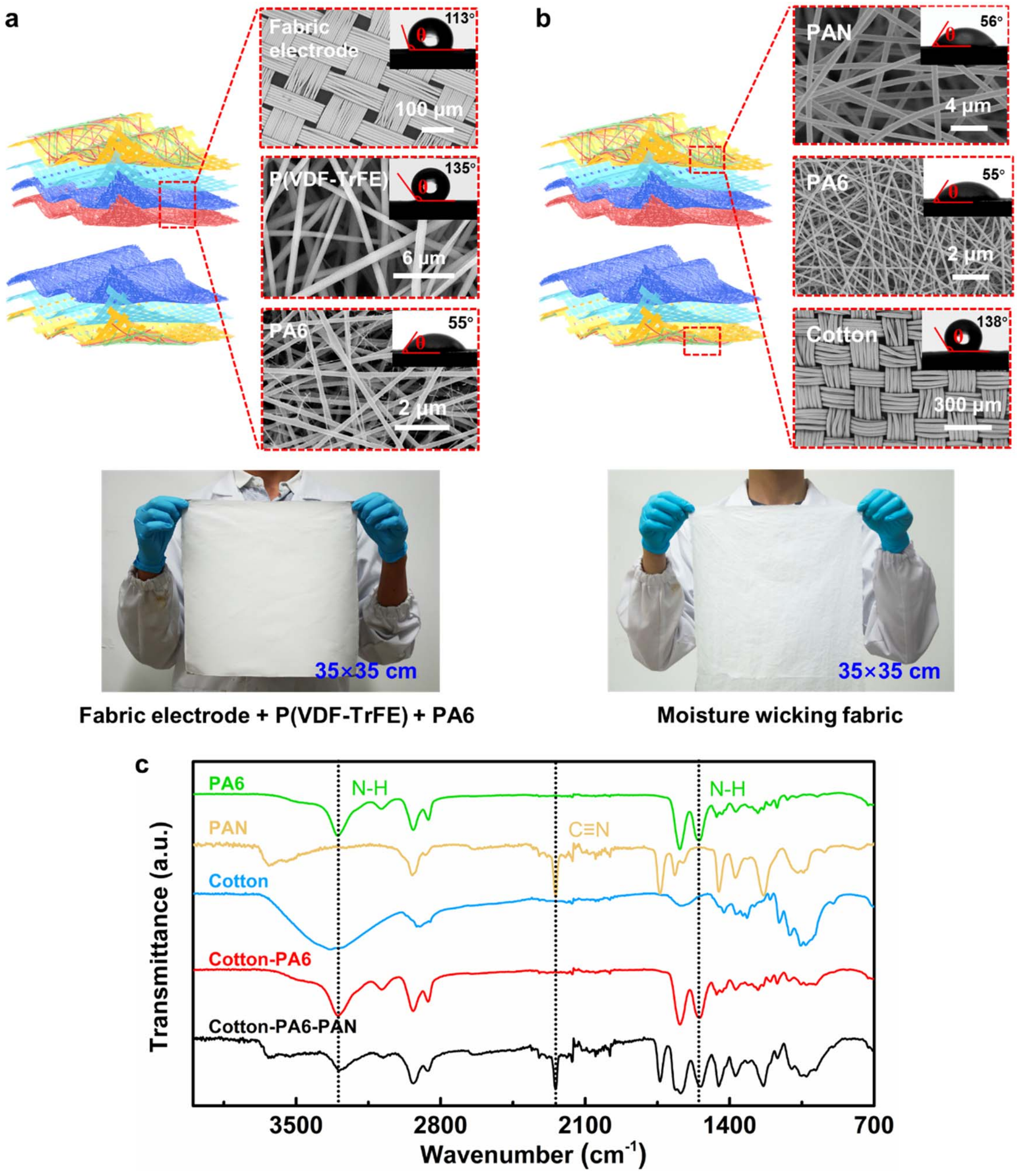

Supplementary Figure 11. Microphotographs, contact angles and infrared characterization of electronics. Physical photos, SEM photos and contact angles of **(a)** Fabric electrode + P(VDF-TrFE) + PA6, **(b)** The moisture-wicking fabric in e-textile. **(c)** ATR-FTIR spectra of the individual layers in the moisture-wicking fabric.

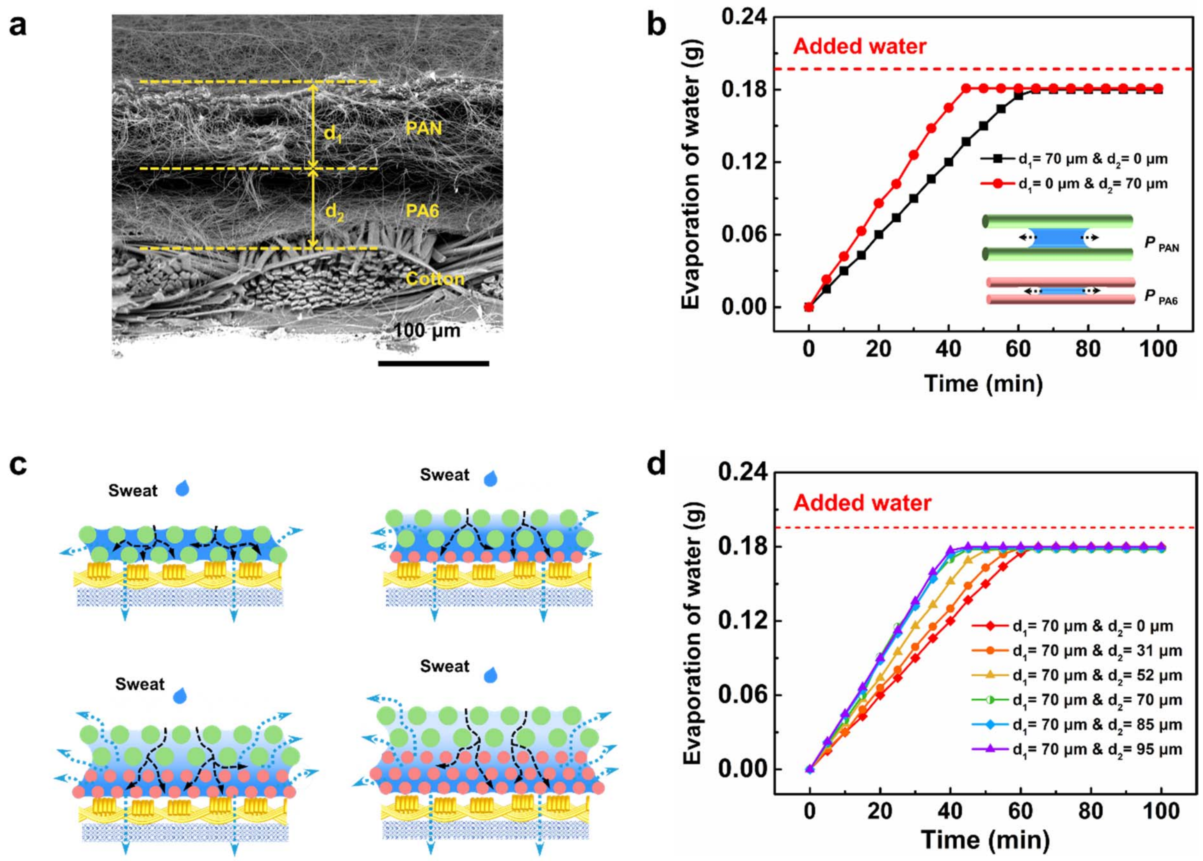

Supplementary Figure 12. Effect of pore size and thickness of fiber layer on water evaporation rate. (a) Cross-sectional SEM images of moisture wicking fabric with trilayered architecture. **(b)** Comparison of water evaporation rates of cotton-PAN and cotton-PA6 fabrics at the same thickness. **(c)** Schematic diagram of water (liquid water: black dotted line, water vapor: blue dotted line) transport at different PA6 layer thicknesses. **(d)** Evaluate the effect of PA6 layer thickness on water evaporation rate of the fabricated textiles. PAN layer thickness was kept constant. The thickness of each fiber layer was controlled by the electrospinning time.

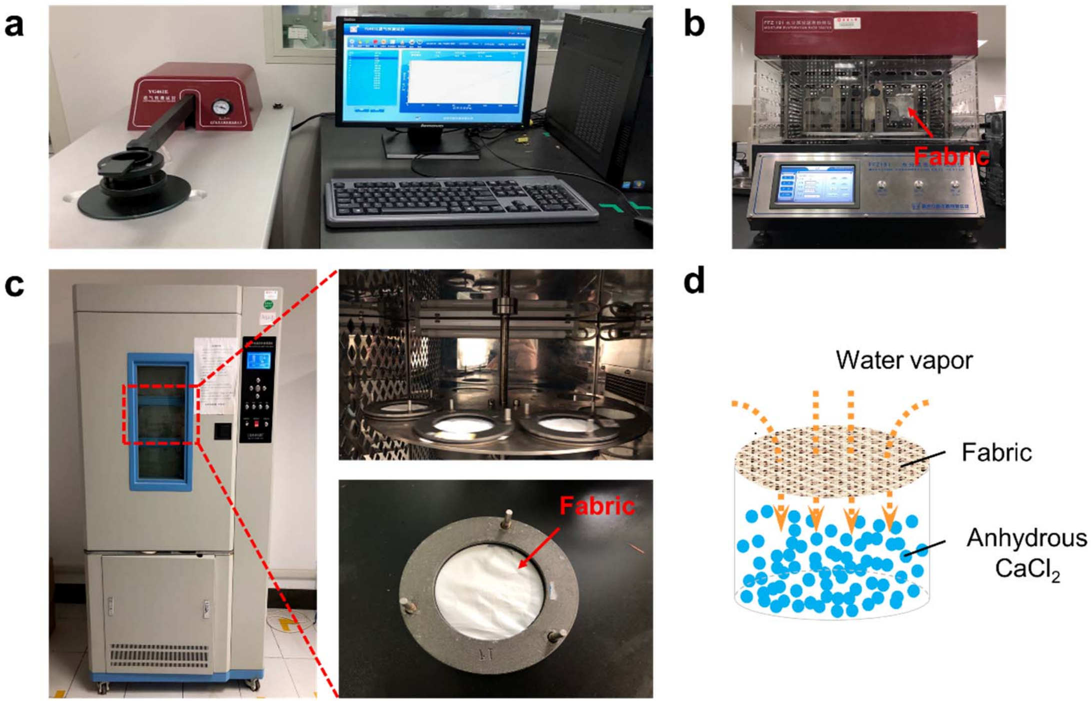

Supplementary Figure 13. Test method of breathability, moisture permeability, water evaporation rate. (a) The fabric permeability to air was measured by the air permeability tester (YG461E, Wenzhou Fangyuan Instrument Co., Ltd., China) followed by GB/T 24218.15-2018 standard. The test was carried out at a pressure of 100 Pa with the machine orifice area of 20 cm^2. **(b)** The water evaporation rate was tested by moisture evaporation rate tester (FFZ191, Wenzhou Fangyuan Instrument Co., Ltd., China) based on GB/T 21655.1−2008 standard. During the test procedure, deionized water (0.2 g) was supplied on the fabric (10 cm × 10 cm), and the weight was recorded every 5 min. **(c)** Water vapor transmission rate test was measure by fabric moisture permeability testing apparatus (YG601H, Ningbo Textile Instrument Factory, China) followed by GB/T 12704.1-2009 standard. **(d)** Desiccant method for testing moisture resistance: 35 g of anhydrous calcium chloride was placed in a moisture permeable cup, and the fabric (diameter 60 mm) was placed on the moisture permeable cup with the test side facing up. The moisture permeable cup mass was weighed every 12 hours in an environment where the temperature was 38 °C and the relative humidity was 86 %.

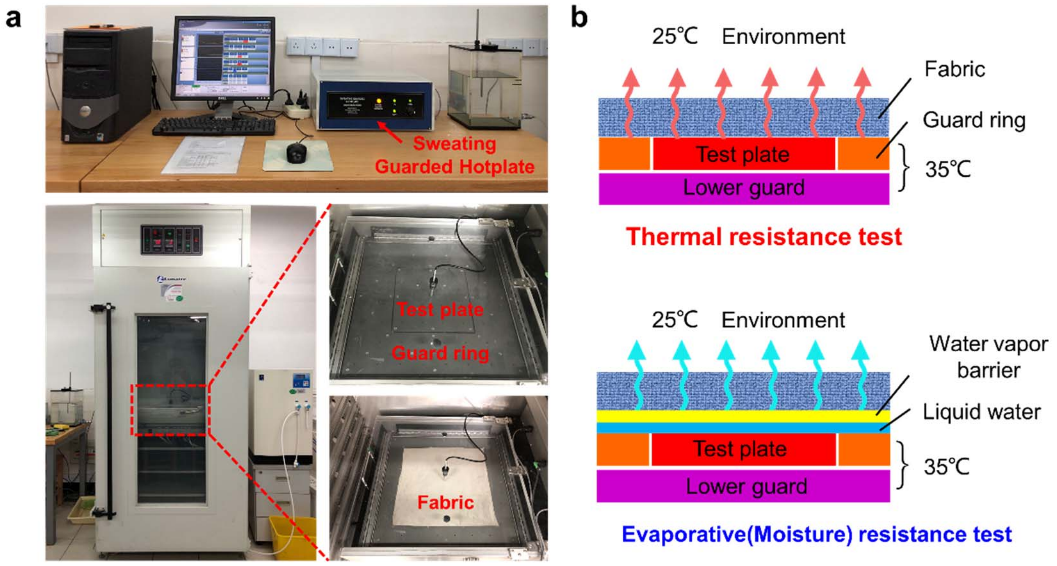

Supplementary Figure 14. Principle of thermal resistance and evaporative resistance test. (a) The thermal and evaporation resistance was assessed according to ASTM-F1868-C standard using a sweating guarded hotplate (SGHP, Northwest Testing Technology Corporation, US). **(b)** Schematic diagram of thermal and evaporative resistance test.

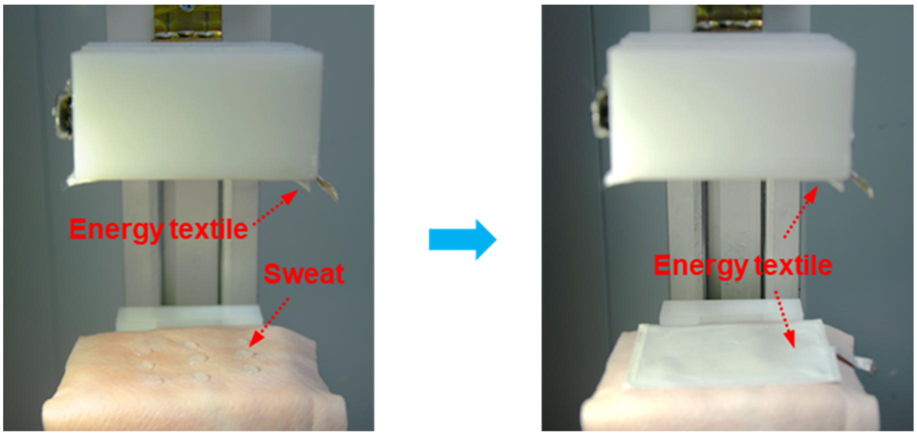

Supplementary Figure 15. Simulation test for different sweating levels of the human body. We prepared 1 wt% sodium chloride (NaCl) solution as simulated sweat. A fixed amount of simulated sweat was aspirated with a pipette and evenly dropped onto the surface of hogskin to simulate the sweating state of the human body. Simulate different

sweating levels of the human body by changing the quality of the sodium chloride solution. The energy fabric was then placed on it and tested for electrical output performance.

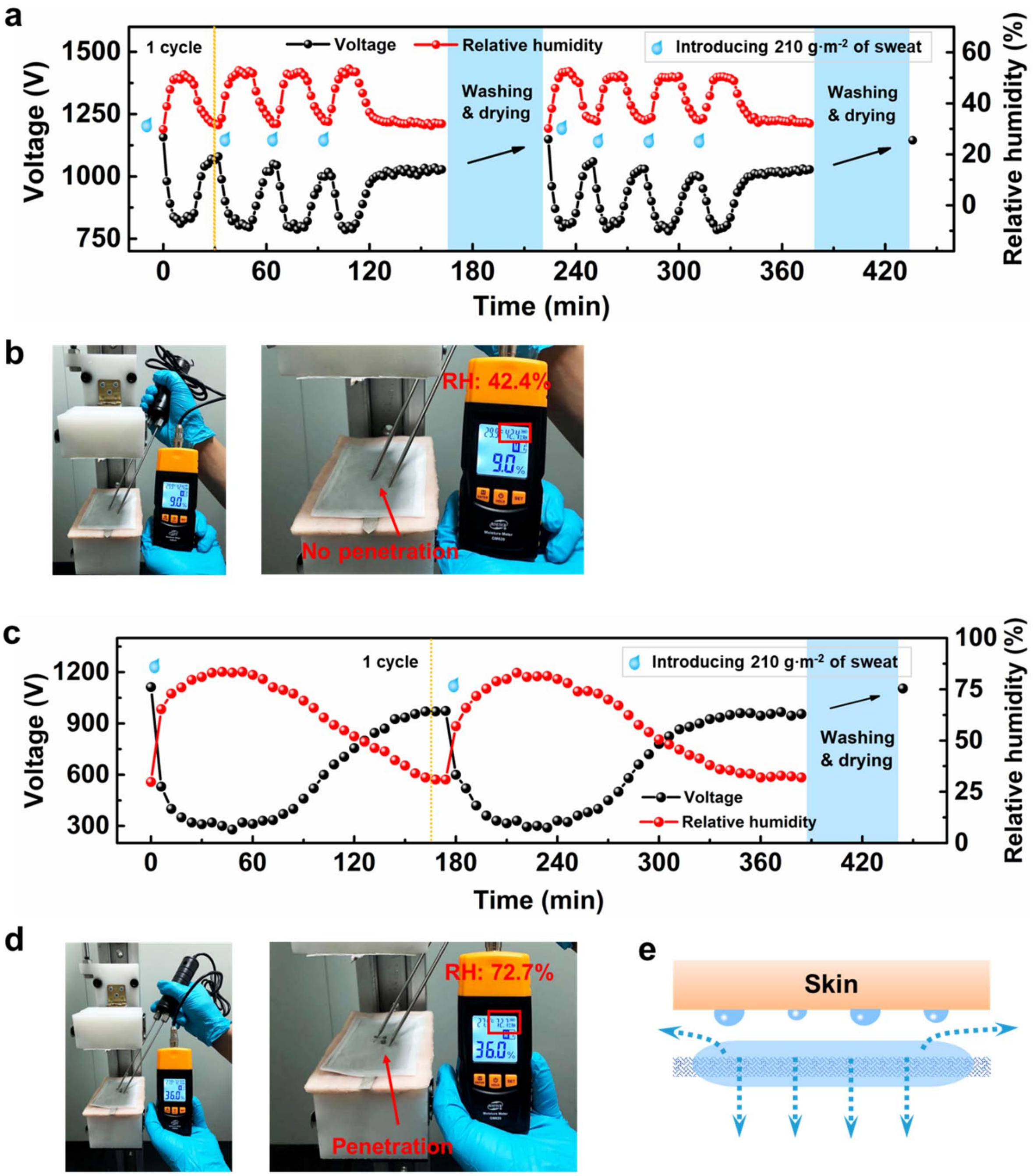

Supplementary Figure 16. Cyclic data of voltage and humidity of e-textiles during moisture wicking. (a) Voltage and surface relative humidity of e-textiles with moisture wicking fabric. **(b)** Test procedure for e- textiles

with moisture wicking layer after introduction of sweat. **(c)** Voltage and surface relative humidity of e-textiles without moisture wicking fabric. **(d)** Test procedure for e- textiles without moisture wicking layer after introduction of sweat. **(e)** E- textiles without moisture wicking layer.

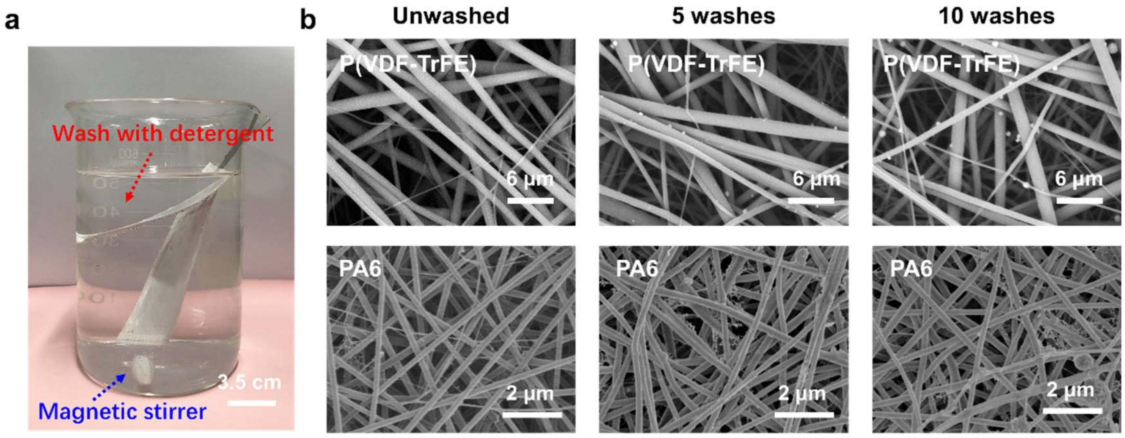

Supplementary Figure 17. Washing durability test of energy textile. (a) Demonstration of the experimental washing environment with household detergent and a magnetic stir bar added. **(b)** Micrographs of P(VDF-TrFE) and PA6 nanofibers without washing, washing 5 and 10 times. It can be seen from the micrographs of P(VDF-TrFE) and PA6 nanofibers that the two nanofiber nonwovens fabrics only have a few impurities remaining on the surface of fibers after 10 times of water washing, and the morphology of fibers does not change significantly. This explains why e-textile did not experience a significant drop in electrical output performance after 10 washes.

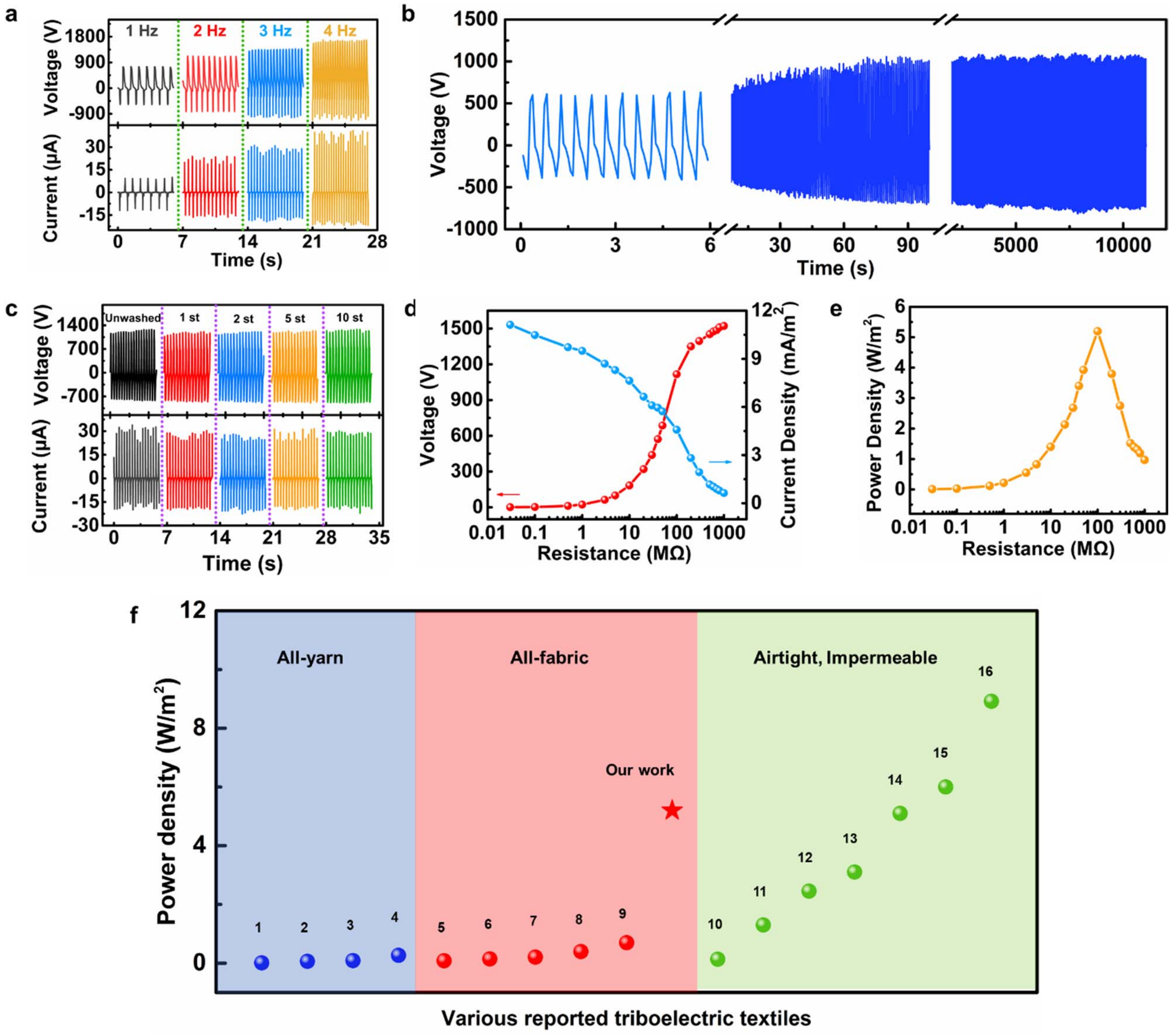

Supplementary Figure 18. Electrical output performance of the e-textiles. (a) Performance of the e-textile at different operating frequencies with a fixed load resistance of 100 MΩ. **(b)** Cyclic stability of e-textiles during continuous work. **(c)** Output voltage (under 100 MΩ) and short-circuit current signals of e-textile after repeated washing. **(d)** Output voltage, current density and **(e)** power density of e-textile measured at different external load resistances varied from 30 KΩ to 1 GΩ at a fixed frequency of 2.5 Hz. **(f)** Comparison with selected important works on triboelectric textiles. 1-16 are the power density data of the all-yarn, all-fabric and the airtight, impermeable triboelectric textiles at the operating frequency below 5 Hz. **[1]** Gong, Wei, et al. Nature communications 10.1 (2019): 868. **[2]** Yu, Aifang, et al. ACS nano 11.12 (2017): 12764-12771. **[3]** Dong, Kai, et al. ACS nano 11.9 (2017): 9490-9499. **[4]** Dong, Kai, et al. Advanced Materials 29.38 (2017): 1702648. **[5]** Qiu, Qian, et al. Nano Energy 58 (2019): 750-758. **[6]** Xiong, Jiaqing, et al. Advanced Energy Materials 7.21 (2017): 1701243. **[7]** Huang, Tao, et al. Nano Energy 58 (2019): 375-383. **[8]** Pu, Xiong, et al. Advanced Materials 27.15 (2015): 2472-2478. **[9]** Guo, Yinben, et al. Nano Energy 48 (2018): 152-160. **[10]** Li, Zhaoling, et al. Nano Energy 53 (2018): 726-733. **[11]** Shen, Jiali, et al. Nano energy 40 (2017): 282-288. **[12]** Yan, Shan, et al. Nano Energy

(2019). Doi:10.1016/j.nanoen.2019.03.021 **[13]** Yu, Bin, et al. Nano Energy 34 (2017): 69-75. **[14]** Xiong, Jiaqing, et al. Nature communications 9.1 (2018): 4280. **[15]** Yu, Bin, et al. Nano Energy 48 (2018): 464-470. **[16]** Tian, Zhumei, et al. Nano Energy 39 (2017): 562-570.

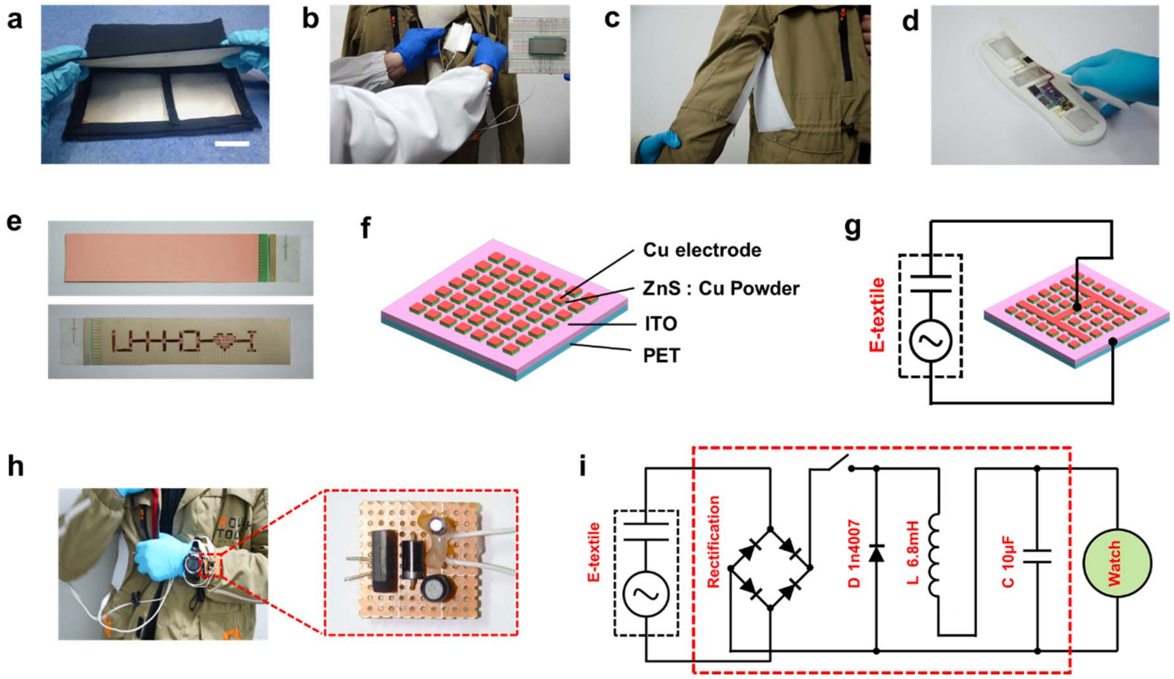

Supplementary Figure 19. Detailed description of the application of the electronics. The e-textile can be perfectly **(a)** sewn together with common textiles or **(b)**, **(c)** directly sewn on existing garments, **(d)** even placed in a sole. The scale bar is 3 cm. E-textile is sewn on the surface of clothes to drive digital electroluminescent lattices. **(e)** Physical photo of the electroluminescent lattice. **(f)** Structural diagram of an electroluminescent. **(g)** A circuit diagram for driving a luminous lattice with e-textiles. An electronic watch was driven by collecting the energy of shoulder movement. **(h)** A power management module [1] was used to improve energy harvesting efficiency of the e-textile and drive an electronic watch in 5 seconds. **(i)** The equivalent circuit diagram for using e-textiles to drive the electronic watch.

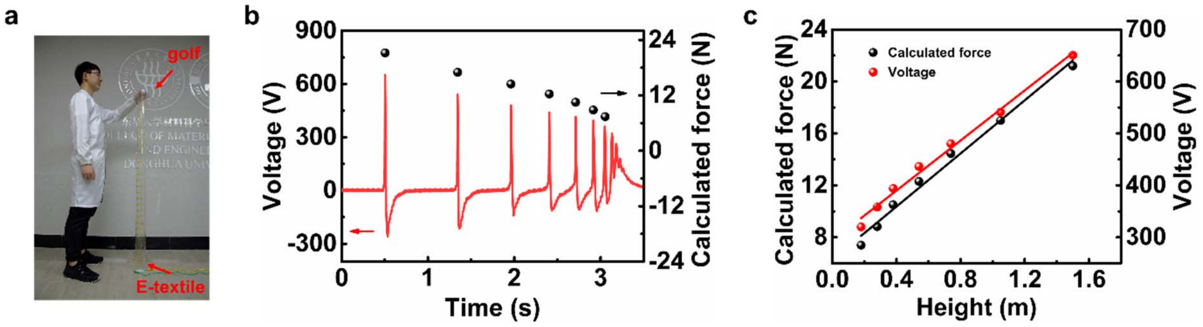

Supplementary Figure 20. The characterization of pressure sensing by free falling impact test. (a) Photo of the free fall impact experiment. **(b)**, **(c)** The relationship between the impact force of golf ball on e-textile and the output voltage of e-textile in the free fall impact test.

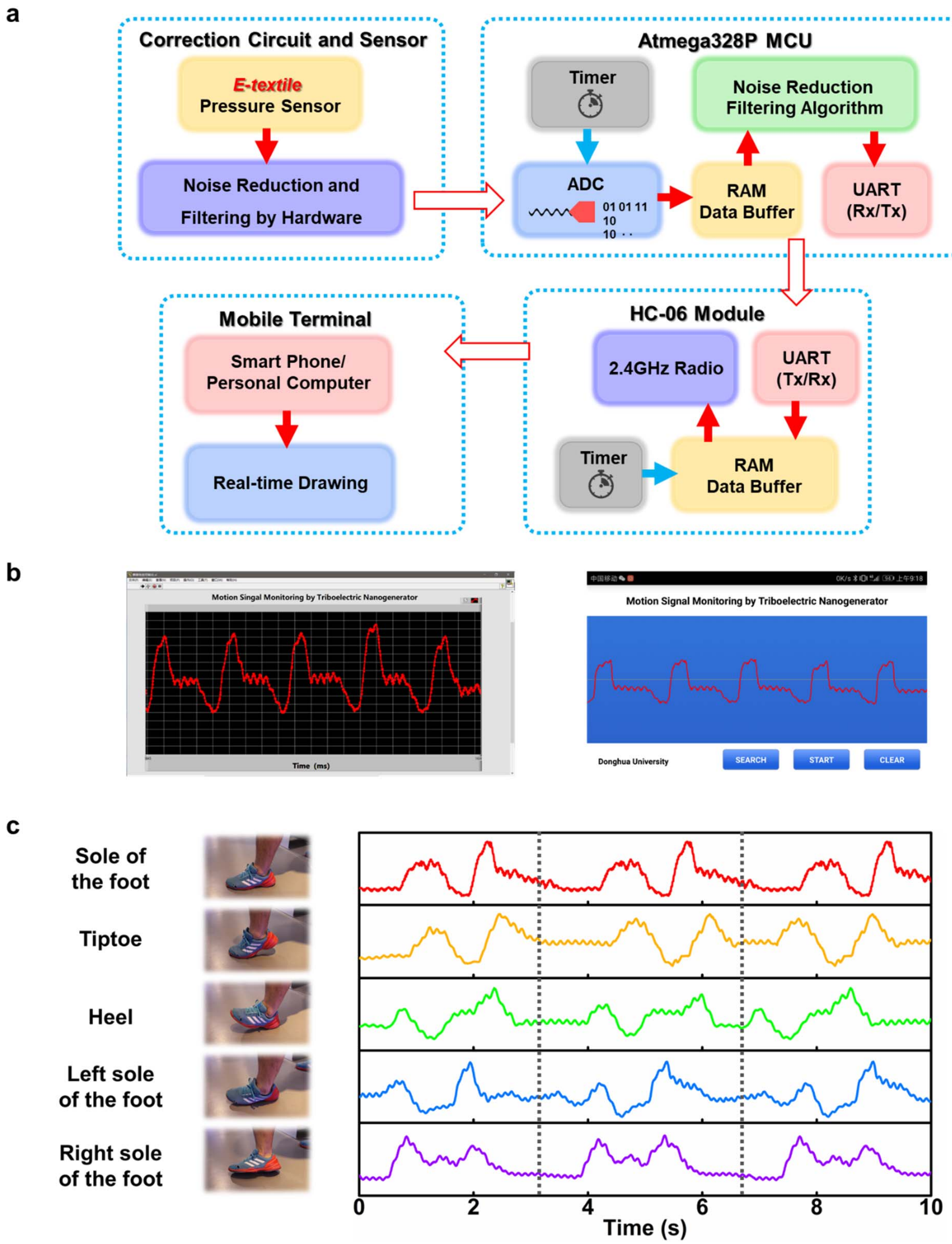

Supplementary Figure 21. The Self-charging, self-sensing monitoring system for capturing human movement. **(a)** A block diagram of the components in self-charging, self-sensing gesture monitoring system. **(b)** The foot motion signals captured by self-powered gesture monitoring system are wirelessly transmitted to personal computers and smartphones. **(c)** The signal output of self-powered gesture monitoring system when monitoring the pressure of different parts of the foot.

Supplementary Tables

Supplementary Table 1. Summary of measurement conditions and electrical parameters of published triboelectric textiles.

	Mode	Tribo-materials	Frequency /Hz	Relative humidity / %	Contact area / cm^{-2}	Voltage / V	Current / μA	Power density / $W \cdot m^{-2}$	Refs.
**All-yarn**	CS	Silicone rubber & Stainless steel	0.5	/	/	60	/	~0.007	[1]
	SE	Polyester & Stainless-steel	/	/	36	~75	~1.2	~0.06	[2]
	SE	Polyester & Stainless steel	3	/	16	~100	~0.72	~0.085	[3]
	CS	PDMS & Stainless steel	3	/	2.25	~35	~0.79	0.263	[4]
**All-fabric**	SE	PET-PVDF-PTFE & Common fabric	3	/	16	112.7	~1.7	0.08	[5]
	SE	PET & Water	/	/	2.25	8	~2.6	0.14	[6]
	FT	PTFE & Common fabric	/	~15	45	~200	~4	0.203	[7]
	CS	Parylene & Ni	0.7	/	25	50	~4.25	0.393	[8]
	CS	PVDF & Silk	2	~30	8	78	~8.2	0.697	[9]
	**CS**	**P(VDF-TrFE) (With two ferroelectric layers) & PA6**	**2**	**~30**	**24**	**1116**	**~13.7**	**5.2**	**Our work**
**Airtight, impermeable**	CS	Polyethersulfone + carbon black + polystyrene & Cellulose acetate	3	/	9	115.2	~1.5	0.13	[10]
	CS	PVDF & Acetate + polyurethane	3	~55	/	345	9.8	1.3	[11]
	CS	PTFE + PVDF + EVOH & Copper	10	/	16	170.5	~14	2.45	[12]
	CS	PVDF & PHBV	2	~20	22	695	~10.1	3.1	[13]
	SE	Hydrophobic cellulose oleoyl ester nanoparticles + black phosphorus & Skin	4	/	49	880	14.7	5.1	[14]
	CS	PVDF & PHBV	2	~25	24.75	~1150	/	6	[15]
	CS	Silicone rubber & Polyester	3	/	25	540	~60	8.92	[16]

CS: Contact-separation, ***SE:*** Single-electrode, ***FT:*** Freestanding triboelectric-layer, ***PDMS:*** Polydimethylsiloxane, ***PET:*** Polyethylene terephthalate, ***PTFE:*** Poly tetra fluoroethylene, ***Ni:*** Niccolum, ***EVOH:*** Ethylene vinyl alcohol copolymer, ***PHBV:*** Poly(3-hydroxybutyrate-co-3-hydroxyvalerate)

Supplementary Table 2. Statistical summary table of relevant parameters in the free fall impact test.

**Number(n)**	**1**	**2**	**3**	**4**	**5**	**6**	**7**	**8**
$\boldsymbol{H}$ **(m)**	1.5	1.05	0.74	0.54	0.38	0.28	0.18	0.13
$\boldsymbol{v}$ **(m s^{-1})**	5.422	4.537	3.808	3.253	2.729	2.343	1.878	1.596
$\boldsymbol{T_D}$ **(s)**	0.55328	0.46291	0.38861	0.33197	0.27848	0.23905	0.19166	0.16288
$\boldsymbol{T_R}$ **(s)**	1.04253	0.87573	0.75063	0.63452	0.54212	0.45872	0.37531	
$\boldsymbol{\Delta T}$	0.02634	0.02421	0.03	0.02407	0.02459	0.02802	0.02077	
$\boldsymbol{T_A}$ **(s)**				0.025				
$\boldsymbol{F}$ **(N)**	20.197	16.997	14.451	12.311	10.507	8.82	7.339	

Drop height $\boldsymbol{H}$ **(m)**, speed $\boldsymbol{v}$ **(m s^{-1})** when landing, drop time $\boldsymbol{T_D}$ **(s)**, recording time $\boldsymbol{T_R}$ **(s)**, average impact time $\boldsymbol{T_A}$ **(s)** and impact force $\boldsymbol{F}$ **(N)**.

Supplementary Notes

Supplementary Note 1. The effect of primary polarization direction (*θ*) and thickness (D_{SFP}) of ferroelectricity on the performance of SFP e-textile.

When the detection between primary polarization and effective electric field ($E_D + E_p$) change from same to opposite, the output voltage and current are significantly improved (Figure S5b). Because the smaller the *θ*, the more difficult it is for dipoles to reach the equilibrium position, resulting in a smaller residual polarization and lower performance. The relationship between output performance and thickness is shown in Figure S5c, and d. The results can be explained by the fact that the increasing of thickness leads to an opposite trend between the amounts of dipoles and internal electric field intensity.

Supplementary Note 2. Depolarization process of electrospun P(VDF-TrFE) nanofiber nonwovens.

In order to prepare an unpolarized P(VDF-TrFE) nanofiber nonwovens that has the same microstructure as the originally polarized nanofiber ferroelectricity, we proposed the idea of **"depolarization"** (Figure S8a)[2]. Here, we treated the electrospun P(VDF-TrFE) nanofiber nonwovens at different temperatures to study the depolarization behavior and microstructure changes (Figure S8b, c, and d). As the heat treatment temperature increases, the d_{33} of P(VDF-TrFE) ferroelectricity decreases continuously. When temperature reaches 190 °C, the nanofibers begin to melt and the microscopic morphology changes, which would change the surface friction behavior and affect the charge transfer process. When the temperature is higher than 180 °C, the d_{33} value does not decrease significantly, which indicates that the heat treatment at 180 °C for 3 h is a suitable depolarization process of P(VDF-TrFE) without significantly changing its microstructure. Figure S8e, f, and g shows the circuit diagram of the d_{33} test. Therefore, the P(VDF-TrFE) obtained by heat treatment at 180 °C for 3 hours can be considered to have completely depolarized. This sample was use in the preparation of unpolarized ferroelectric (UP) e-textile for control experiments.

Supplementary Note 3. Comparison of electrical properties of UP, SFP and DFP e-textiles to verify the tribo-ferroelectric synergistic mechanism.

Figure S9a shows the process of charges accumulation in three e-textiles step by step. The UP e-textile reaches a maximum value of ~106 $\mu C \cdot m^{-2}$ only after 1100 cycles operation, while the SFP and DFP e-textiles increase with a much bigger slope and reach the maximum value of ~161 $\mu C\ m^{-2}$ after 2800 cycles and ~320 $\mu C\ m^{-2}$ after 6600 cycles. Similarly, with the continue of contact-separate processes, the DFP e-textile has the fastest growth rate and the highest saturation current (~33 μA).

The current growth rate and saturation current (~16 μA) of SFP e-textile are higher than those of UP e-textile (~10 μA). In addition, during the contact-separation process, we also tested the surface potential of tribo-negative material (P(VDF-TrFE)). It was detected every 10 seconds, and the distance between probe and the textile was fixed at 1.5 cm (Figure S9c). In 600 seconds, the surface potential of P(VDF-TrFE) in UP e-textile increased from -0.3 kV to -1.05 kV, while the SFP and DFP textiles increased from -0.34 kV and -0.29 kV to -1.91 kV and -3.1 kV, respectively. It visually shows the accumulation of triboelectric charges step by step. And the surface charges accumulation rate and charge density at the friction interface were significantly improved due to the tribo-ferroelectric synergistic enhancement mechanism. As shown in Figure S9d, the output voltage results have similar trend.

Supplementary Note 4. Detailed description of the tribo-ferroelectric synergistic mechanism in DFP e-textile.

The e-textile consists of two nanofiber nonwovens P(VDF-TrFE) and PA6 with opposite tribo-polarity for contact electrification, Ni-Cu fabric electrode for charge induction. The P(VDF-TrFE) nanofibers also act as a polymer ferroelectricity (defined as inner/outer ferroelectric layers) for constructing tribo-ferroelectric synergistic enhancement effect (Figure S10). The in-situ polarization effect of electrospinning P(VDF-TrFE) is shown in Figure S10a. When PA6 and P(VDF-TrFE) are in contact, they will acquire net opposite charges on their surfaces (b). Once PA6 is separated from P(VDF-TrFE), electrons flow from fabric electrode II to fabric electrode I (c). Meanwhile, the induced potential between two charged surfaces will result in a second polarization of P(VDF-TrFE) ferroelectricity. The polarization of P(VDF-TrFE) ferroelectricity will keep enhanced until the distance between two tribo-polarity materials reaches maximum (d). As the separation distance decreases, the polarization of P(VDF-TrFE) ferroelectricity will gradually decrease until the PA6 contact with P(VDF-TrFE) again (e). Due to dielectric hysteresis [3-5], however, the polarization inside the inner and outer ferroelectric layers will not fully diminish, and the residual built-in dielectric polarization will act as positive and negative charge trap to enhance the capability of capturing charges during contact electrification (f).

Supplementary Note 5. Effect of PA6 layer thickness on water evaporation rate of moisture wicking fabric.

To evaluate the effect of PA6 layer thickness on water evaporation rate of the fabricated textiles, top layer (PAN) thickness and the volume of sweat (200 μL) were kept constant. The increase in the thickness of PA6 layer enriches the mass of the layer, thereby enhancing the water absorption capacity of moisture wicking fabric and also giving rise to the wettability gradient between two layers (From

light blue to dark blue) [6, 7]. Since the PA6 layer has a faster penetration and spreading driving force (Figure S12b) than PAN layer, more sweat enrichment in PA6 layer will be more conducive to evaporation. As shown in Figure S12c and d, with the increase of PA6 layer thickness (from 0 μm, 40±6 μm, 70±8 μm to 85±5 μm), the water evaporation rate of moisture wicking fabric increases. Since the thickness of 85±5 μm of PA6 layer is enough to pull out almost all water from the top (PAN) layer, the water evaporation rate gradually reaches saturation as the thickness of PA6 further increases (from 85±5 μm, 95±9 μm to 100±6 μm).

Supplementary Note 6. Thermal resistance and evaporative resistance test methods and calculation formulas for functional textiles [8].

Thermal resistance test: To simulate the skin of human body and its surrounding area, the SGHP consists of three independently controlled heating zones: test plate, guard ring and lower guard. Each zone is heated to the same temperature (typically 35 °C, close to human skin temperature) to eliminate heat transfer between the different zones (Figure S14b). Therefore, all heat loss will only pass through the fabric to surrounding environment (typically 25 °C). The thermal energy (Q) required to maintain the set temperature for each zone is measured. Thermal resistance can be expressed as

$$R_{ct} = \frac{T_{sikn} - T_{amb}}{Q/A} \quad (1)$$

where T_{skin} is the test plate temperature (°C); T_{amb} is the ambient air temperature (°C); Q/A is the test plate heat flow (W m^{-2}).

Evaporation resistance test: A vapor barrier layer, such as fiberglass paper, was placed between the test plate and the sample to keep the liquid water from wetting the sample. This method allows water vapor to pass through the vapor barrier while the liquid can't pass through (Figure S14b). Similarly, the evaporation resistance can be calculated by measuring the water vapor pressure on the upper and lower sides of the sample, expressed as

$$R_{et} = \frac{P_{sikn} - P_{amb}}{Q/A} \quad (2)$$

$$P_{amb} = RH \cdot 133.3 \cdot 10exp[8.10765 - (1750.29/(235 + T_{amb}))] \quad (3)$$

$$P_{skin} = 133.3 \cdot 10exp[8.10765 - (1750.29/(235 + T_{skin}))] \quad (4)$$

where P_{amb} is the vapor pressure (Pa) of ambient water; P_{skin} is the vapor pressure (Pa) of water above the test plate; RH is the ambient relative humidity (%).

Supplementary Note 7. Cyclic test of electrical output and surface relative humidity of e-textiles during multiple moisture wicking.

As shown in Figure S16a, after introducing 210 g m^{-2} of sweat onto the surface of hogskin, the relative humidity of friction material gradually increased from 30 to ~50 %, and lasted for about 10 min at 50 % RH. Correspondingly, the output voltage (under 100 MΩ load) of e-textile was gradually reduced from 1110 to ~800 V, and maintained for about 10 min (the voltage data in Figure 4d is obtained at this stage). Subsequently, the relative humidity of friction material gradually decreased to 30 % and reached equilibrium, and the corresponding voltage also raised to equilibrium. So far, the moisture wicking process have undergone a cycle, lasting about 30 minutes. For e-textile without moisture wicking fabric, the relative humidity of friction surface raised from 30 to 83%, and maintained for about 60 minutes at a high relative humidity. The voltage also had a similar trend at this stage, from 1100 to ~300 V. The whole cycle time is about 160 minutes as shown in Figure S16c. At the end of each moisture wicking process, the output voltage of the electronic textile can be gradually recovered, but less than the initial output voltage. This may be caused by the influence of residual salt such as sodium chloride (NaCl) in e-textile on output voltage. It is noteworthy that after washing and drying, the output voltage of e-textile can return to initial state.

Supplementary Note 8. Description of the washing test.

3 mL of laundry detergent, 600 mL of deionized water and energy textile were first added to the beaker, and magnetic stirring was performed at 600 rpm to simulate the washing environment. Wherein e-textile is placed directly into the wash solution without any packaging. The wash cycle time was 30 min. Finally, the e-textile was naturally dried for subsequent electrical output measurements (Figure S17).

Supplementary Note 9. Interpretation of the comparison method of output power density in triboelectric textiles.

We systematically sorted out the electrical performance output and measurement conditions of the energy textiles to the best our knowledge. These triboelectric textiles are mainly divided into three categories: All-yarn (breathable), All-fabric (breathable) and Airtight (impermeable) as shown in Figure S18f, and Table S1 . It should be noted that the voltages and currents listed in table are the corresponding outputs of peak power density.

Supplementary Note 10. Free falling impact test proves that e-textile has certain pressure sensing characteristics.

In order to prove that e-textile has certain pressure sensing characteristics, we used a golf ball with a diameter of 43 mm and a mass of 50 g for free fall impact experiments to simulate the pressure on e-textile when walking (Figure 20a). We use the Keithley 2657A to test the voltage signal produced by each impact of e-textile and use a digital camera to record the time and height of each free fall of the golf ball. The magnitude of the force each time the golf ball hits e-textile is calculated using the formula

$$\frac{1}{2}mv^2 = mgh \quad (5)$$

$$m(v_{n+1} + v_n) = F_n T_A \quad (6)$$

Related experimental data are shown in Table S2. Figure S20b and c show that the calculated value of the impact force of e-textile has a good linear relationship with the output voltage signal.

Supplementary Note 11. Interpretation of signal processing, real-time mapping, and foot pressure sensing in self-charging and self-sensing gesture monitoring system.

The analog signal processing and wireless transmission unit consists of a processor chip Atmega328P and a wireless transmission module HC-06, mainly adopting analog-to-digital conversion (ADC), digital filtering noise reduction and transmission technology. When processed electrical signals are transmitted to Atmega328P, they are converted to digital signals and filtered by an algorithm. Then, the HC-06 establishes wireless data transmission in 2.4G Hz band with external devices such as a smart phone and personal computer (Figure S21a). After mobile terminal receives the data, it performs real-time data drawing (Figure S21b). In addition, the data collected by mobile terminal can also be transmitted to the Internet and cloud mega data. It is expected to realize online real-time monitoring feedback, online diagnosis and the other functions.

Supplementary References

1. Xi, F. et al. Universal power management strategy for triboelectric nanogenerator. *Nano Energy* **37,** 168-176 (2017).
2. Lei, T. et al. Electrospinning-induced preferred dipole orientation in PVDF fibers. *J. Mater. Sci.* **50,** 4342-4347 (2015).
3. Fumagalli, L. et al. Anomalously low dielectric constant of confined water. *Science* **360,** 1339-1342 (2018).
4. Zhang, C., Gygi, F. & Galli, G. Strongly anisotropic dielectric relaxation of water at the nanoscale. *J. Phys. Chem. Lett.* **4,** 2477-2481 (2013).
5. Hill, N. E. Interpretation of the dielectric properties of water. *Transactions of the Faraday Society* **59,** 344-346 (1963).
6. Miao, D., Huang, Z., Wang, X., Yu, J. & Ding, B. Continuous, spontaneous, and directional water transport in the trilayered fibrous membranes for functional moisture wicking textiles. *Small* **14,** e1801527 (2018).
7. Babar, A. A. et al, Breathable and colorful cellulose acetate-based nanofibrous membranes for directional moisture transport. *ACS Appl. Mater. Interface* **10,** 22866-22875 (2018).
8. ASTM F 1868. Standard test method for thermal and evaporative resistance of clothing materials using a sweating hot plate. (2014).
