# Supplementary material for: All-fiber tribo-ferroelectric synergistic electronics with high thermal-moisture stability and comfortability
Source: Nat Commun. 2019 Dec 5;10:5541. doi: 10.1038/s41467-019-13569-5 (PMC6895236; doi:10.1038/s41467-019-13569-5)
Supplement: Supplementary file 3 — Description of Additional Supplementary Files [file 41467_2019_13569_MOESM3_ESM.pdf]

Description of Additional Supplementary Files

File Name: Supplementary Movie 1
Description: Simulating the wetting and diffusion behavior of human sweat on the e-textile (the surface of the moisture-wicking fabric).

File Name: Supplementary Movie 2
Description: Common wearable electronic devices are driven by e-textile in various scenarios.

File Name: Supplementary Movie 3
Description: The self-charging, self-sensing system captures the foot posture and transfers it to smartphone and computer for drawing in real time
